# Bridging molecular advancements and clinical challenges in pediatric oncology

**DOI:** 10.1007/s12519-024-00870-7

**Published:** 2024-12-19

**Authors:** Michaela Kuhlen, Michael C. Frühwald

**Affiliations:** Pediatric and Adolescent Medicine, Faculty of Medicine, University of Augsburg, Stenglinstr. 2, 86156 Augsburg, Germany

Pediatric oncology has always been a dynamic and ever-evolving field dedicated to understanding and combating cancers in children and adolescents. While survival rates for many pediatric malignancies have improved significantly especially until the late 1990s, [1–3] a closer examination reveals areas where progress has since stagnated, particularly in solid tumors and rare cancers [4, 5]. We herein explore two critical issues in pediatric oncology: the challenges in translating molecular insights into effective treatments for solid tumors and the urgent need for advancing practice-changing research on rare pediatric malignancies.

## Advances and limitations of molecular precision medicine

The genomic revolution has provided a wealth of insights into the biology of pediatric cancers [6–9]. Next-generation sequencing (NGS) and other molecular diagnostic tools allow for into-depth tumor profiling, making the identification of specific genetic drivers of malignancies possible, almost routine [10, 11]. In principle, these advances herald an era of precision medicine, where targeted therapies are tailored to individual patients based on tumor’s unique molecular characteristics.

However, the promise of molecular medicine has not yet been fully realized in pediatric oncology, particularly for solid tumors. While genomic studies reveal potentially actionable findings in up to 20%–40% of cases, the clinical translation of these discoveries remains limited [12–14]. Several factors contribute to this gap. Many targeted therapies designed for adult malignancies are either unavailable or inadequately tested in children, leaving pediatric oncologists reliant on off-label use or experimental approaches. The intrinsic complexity and heterogeneity of solid tumors often preclude the identification of universally effective targets. Even when promising agents are identified, logistical hurdles—such as patient selection, the timing of molecular testing, and data interpretation—further complicate their clinical application. Children are no small adults; the specifics of the developing organism must always be considered. A fact that deters pharmaceutical companies from investing into novel drugs due to the inherent risk of such an approach in a highly vulnerable population.

Accordingly, despite advances in precision diagnostics, survival rates for children with advanced or recurrent solid tumors have seen little improvement [3]. This disconnect underscores the need for a more robust framework to integrate molecular findings into therapeutic strategies. Tools such as molecular tumor boards and liquid biopsy monitoring will aid in this endeavor, enabling real-time of follow-up of tumor dynamics, thus facilitating adaptive treatment approaches.

## Challenges of rare pediatric tumors

Rare pediatric cancers, such as adrenocortical carcinoma or rhabdoid tumors, exemplify the challenges we face [15, 16]. These malignancies often evade the spotlight of mainstream research due to their low incidence, yet their impact on affected patients and families is profound. Outcomes for these tumors remain dismal, with few therapeutic advances in recent years [17, 18].

Investigating rare pediatric cancers encounters numerous obstacles, such as the difficulty of recruiting sufficient patient numbers into robust controlled clinical trials and a lack of dedicated funding. These hurdles call for innovative approaches to study design, such as collaborative networks that pool data and samples across institutions. Initiatives like the INFORM registry demonstrate the potential of such efforts by identifying molecular targets and facilitating access to investigational therapies [13, 19]. However, much more remains to be done to ensure equitable access to research and treatment opportunities for children with rare cancers.

## Call to action

The path forward in pediatric oncology must be rooted in collaboration, innovation, and advocacy. Multidisciplinary teams—comprising pediatric oncologists, pediatric radiologists, pediatric surgeons, neurosurgeons, geneticists, pathologists, and psychosocial experts—are critical to navigating the complexities of care and ensuring that no child is left behind. At the same time, policymakers and funding organizations must prioritize pediatric oncology as an area of critical need, providing the resources necessary to advance research on rare and challenging cancers.

Pediatric oncology has been at the forefront of pioneering groundbreaking therapies like blinatumomab and CAR T-cell therapy, positioning itself as a potential catalyst in the progression of cancer treatment [20, 21].

To fully leverage the potential of molecular medicine, we must also invest in the next generation of clinical trials, specifically designed for pediatric populations. These trials should evaluate not only the safety and efficacy of targeted therapies but also their long-term impact on survivors’ quality of life and immediate consequences on affected families. Furthermore, incorporating cutting-edge diagnostic tools, such as liquid biopsy, into routine care will enhance our ability to deliver precision treatment, particularly for relapsed or refractory cases.

## Conclusions

The field of pediatric oncology stands at a crossroads, with unprecedented opportunities for progress tempered by significant challenges. By addressing the barriers to implementing molecular advances and prioritizing research on rare cancers, we should pave the way for a future where every child diagnosed with cancer has access to the best possible care. It will be only through a concerted, multidisciplinary effort that we will fulfill the promise of personalized medicine in pediatric oncology, improving outcomes and offering hope to children and families worldwide.

## Data Availability

Not applicable.
